# Integrative analysis of the common genetic characteristics in ovarian cancer stem cells sorted by multiple approaches

**DOI:** 10.1186/s13048-020-00715-7

**Published:** 2020-09-25

**Authors:** Xiaoxiao Zhang, Yue Su, Xue Wu, Rourou Xiao, Yifan Wu, Bin Yang, Zhen Wang, Lili Guo, Xiaoyan Kang, Changyu Wang

**Affiliations:** 1 Department of Gynecology and Obstetrics, Tongji Hospital, Tongji Medical College, Huazhong University of Science and Technology, 1095 Jiefang Anv., Wuhan, 430030 Hubei China; 2 Department of Gynecology and Obstetrics, The Central Hospital of Wuhan, Wuhan, Hubei China

**Keywords:** Bioinformatic analysis, Ovarian cancer, Ovarian cancer stem cells (OCSCs), Cancer stem cells markers, Differentially expressed genes (DEGs), Therapeutic targets

## Abstract

**Background:**

Ovarian cancer is the second fatal malignancy of the female reproductive system. Based on the cancer stem cell (CSC) theory, its poor prognosis of ovarian cancer attributed to tumor recurrence caused by CSCs. A variety of cell surface-specific markers have been employed to identify ovarian cancer stem cells (OCSCs). In this study, we attempted to explore the common feature in ovarian cancer stem cells sorted by multiple approaches.

**Methods:**

We collected the gene expression profiles of OCSCs were from 5 public cohorts and employed R software and Bioconductor packages to establish differently expressed genes (DEGs) between OCSCs and parental cells. We extracted the integrated DEGs by protein-protein interaction (PPI) network construction and explored potential treatment by the Cellminer database.

**Results:**

We identified and integrated the DEGs of OCSCs sorted by multiple isolation approaches. Besides, we identified OCSCs share characteristics in the lipid metabolism and extracellular matrix changes. Moreover, we obtained 16 co-expressed core genes, such as *FOXQ1, MMP7, AQP5, RBM47, ETV4, NPW, SUSD2, SFRP2, IDO1, ANPEP, CXCR4, SCNN1A, SPP1* and *IFI27* (upregulated) and *SERPINE1, DUSP1, CD40,* and *IL6* (downregulated). Through correlation analysis, we screened out ten potential drugs to target the core genes.

**Conclusion:**

Based on the comprehensive analysis of the genomic datasets with different sorting methods of OCSCs, we figured out the common driving genes to regulating OCSC and obtained ten new potential therapies for eliminating ovarian cancer stem cells. Hence, the findings of our study might have potential clinical significance.

## Background

Ovarian cancer is the second most lethal gynecologic malignancy in women around the world [1]. Debulking surgery and platinum-based chemotherapy results in complete response in 70% of patients, most will relapse or even succumb to chemoresistance [2]. Significant progress in maintenance therapy has been seen by combination with poly (ADP-ribose) polymerase inhibitors, which have been approved in disease recurrence and a first-line setting among women with BRCA1/BRCA2 mutations [1]. Tumor recurrence has been attributed to suboptimal resection and the presence of residual chemo-resistant OCSCs [2, 3].

Over the years, multiple biomarkers have been identified exclusively or co-expressed in OCSCs and have been explored for their unique functions in tumorigenesis [4]. Several studies have contributed to the isolation and identification of OCSCs. Spheroids’ formation in cancer stem cell culture has been recognized as the first commonly used approach [5, 6]. With the Hoechst 33342 dye, Side population (SP), have overexpressed several members of ABC transporters and exhibited some characteristics of CSCs, are collected [7, 8]. Based on cell surface markers, CD44, CD117, and CD133 etc., OCSCs have been successfully identified and isolated [9–11]. The activity of ALDH1 has been widely used in the identification of stem/progenitor cells or CSCs. Cells expressing high levels of ALDH1 can be identified by ALDEFLUOR assay and isolated by the ALDH1 antibody [10, 12, 13].

CSCs have generally been attributed to the heterogeneity of tumors. Stem cell-associated heterogeneity resulted from intrinsic tumor plasticity can be shaped by the microenvironment [14]. Many abnormal signaling pathways of CSC play a vital role in its maintenance, survival and metastasis, including Hedgehog, Notch and Wnt/β-catenin pathways, carcinogenic cascades such as PI3K/AKT, TGF-β, EGFR, JAK/STAT or NF-κB as well as transcriptional regulators such as OCT4, Nanog, YAP/TAZ and Myc [3]. OCSCs identified by the different approach has shown different mechanisms for maintaining cancer stem-like properties. They may share the same biomarkers as well as biological characteristics. This has led to an increasing interest in elucidating the underlying mechanism of OCSCs identified by different methods.

In this study, we downloaded five original microarray datasets, namely, GSE82305 [13], GSE28799 [5], GSE53759 [15], GSE94358 [16], and GSE33874 [17], from the GEO database, and these datasets contain a total of 45 samples, including 21 OCSCs samples and 24 parental cancer cell samples. We used R language software to standardize all the datasets and to get DEGs. The ‘RobustRankAggreg’ package was subsequently used to integrate the results and obtain integrated differentially expressed genes (DEGs). By function and pathway analysis, we identified OCSCs share characteristics in the lipid metabolism and extracellular matrix changes. Combined with WGCNA and PPI network, we identified the hub genes of OCSC and obtained 16 co-expressed core genes, such as *FOXQ1, MMP7, AQP5, RBM47, ETV4, NPW, SUSD2, SFRP2, IDO1, ANPEP, CXCR4, SCNN1A, SPP1* and *IFI27* (upregulated) and *SERPINE1, DUSP1, CD40,* and *IL6* (downregulated). Based on the comprehensive analysis of the genomic datasets with different markers of OCSCs, we figured out the common driving signal pathways to regulating OCSCs. Finally, we obtained ten new potential therapies for the elimination of ovarian cancer stem cells.

## Methods

### Data procession

The gene expression profiles of OCSCs (GSE82305, GSE28799, GSE53759, GSE94358) were downloaded from the Gene Expression Omnibus (GEO) database (https://www.ncbi.nlm.nih.gov/geo/). The dataset information is shown in Table 1. Data adjustments included data filtering, normalization, and ID transformation. Each dataset was then normalized using the normalize Between Arrays function in the R package ‘limma’ (http://www.bioconductor.org/). The 288 OV RNA-seq transcriptome data were download from the UCSC Xena (https://xenabrowser.net/) and were calculated as log2(x + 1) transformed RSEM normalized counts. The 88 normal ovarian samples were obtained through the Genotype-Tissue Expression (GTEx) [18] and were calculated as log2(x + 1) transformed RSEM normalized counts.

**Table 1:** Details of datasets of OCSCs in the GEO

GEO accession	Platform	Organism	Details
GSE82305	GPL10558	*Homo sapiens*	SKOV3 aldefluor(+)/aldefluor(−)
GSE53759	GPL6244	Homo sapiens	IGROV-1 spheroids/non-spheroids
GSE28799	GPL570	Homo sapiens	OVCAR3 spheroids/adherent
GSE94358	GPL570	Homo sapiens	spheroids/adherent
GSE33874	GPL570	Homo sapiens	Side population/main population

### Integration of microarray data

The R package ‘limma’ was used to test DEGs in each dataset. Genes with an adjusted *P*-value < 0.05 and |log fold change (FC)| > 1 were considered DEGs. The DEGs in the four datasets were integrated using the R package ‘RobustRankAggreg’ (http://www.bioconductor.org/). The integrated upregulated and downregulated DEG lists were saved for subsequent analysis.

### Weighted gene co-expression network analysis

The variant genes in the GSE33874 dataset were constructed to an approximate scalefree fundamental gene co-expression network using the R package ‘WGCNA’ [19]. Genes with a high correlation were clustered and the network modules were generated using the topological overlap measure (TOM). The color bars correspond to the clusters of genes can be seen as the gene module. The threshold of the co-expression module was set as *p* < 0.05.

### Function and pathway analysis

The gene ontology (GO) annotation and Kyoto Encyclopedia of Genes and Genomes (KEGG) pathway enrichment analyses of the integrated DEGs were performed using the DAVID 6.8 database (https://david.ncifcrf.gov/). GO terms were classified in three categories: biological process (BP), cellular component (CC), and molecular function (MF). The term with highest −log10qValue was determined the most significant enrichment. Q-values below 0.05 (q < 0.05) were considered significant. The visualization of the GO and KEGG pathway enrichment analyses was performed using R 3.6.3 software.

### Protein-protein interaction (PPI) network construction and hub gene selection

The PPI network of the integrated DEGs was analyzed with the STRING database (http://string-db.org/) and visualized using Cytoscape 3.8.0 software. The plug-in molecular complex detection of Cytoscape was subsequently applied to construct the subnetwork for further analysis. The top 3 cluster, with the default parameters “false Degree Cutoff = 2”, “Node Score Cutoff = 0.2”, “K-Core = 2” and “Max.Depth from Seed = 100”, was saved and listed in Table 3. Genes in a significant module of WGCNA were analyzed, and the top 3 subnets were listed in Table 2.

**Table 2:** The top 3 subnets of genes in the blue module

Cluster	Score (Density*#Nodes)	Nodes	Edges	Node IDs
1	21	21	210	WSB1, SPSB1, RNF14, MYLIP, RNF114, RNF19B, UBE2D1, KLHL21, FBXL7, SMURF2, NEDD4L, GLMN, UBE2L6, ANAPC4, RNF19A, HERC2, WWP1, KBTBD7, UBE2F, TRIM32, RNF144B
2	14.174	24	163	IL18, CSF2, PTGER3, GNB4, GNG11, ICAM1, OXGR1, ITGAM, CSF3, ACKR3, CCR1, IL6R, CXCL3, HTR1D, TLR2, C3, IL1R1, CXCL1, SSTR2, ADRA2B, BDKRB1, VCAM1, IL15, CD44
3	11.234	48	264	NR4A1, TRAF1, ATF3, NFKB1, TNFAIP3, CCK, NFKBIA, IKBKB, CD83, TNFRSF10B, SERPINB2, IL6, ADRB2, AVPR1B, IRAK2, AGTR1, GADD45B, KITLG, P2RY1, HRH1, FOSB, RIPK1, WNT5A, REL, BTG2, STAM, JUN, RIPK2, STON1, PIK3R3, NR4A2, DUSP1, LGALS3, PACSIN2, VAMP2, CD40, DNAJC6, ZFP36, TSLP, IRF1, LRP2, PIK3R1, SERPINE1, JUNB, FOSL1, SELE, NECAP2, TFRC

### Gene expression in immune subtypes

A new immune classification of solid tumors has identified six immune subtypes (C1-C6) [20]. Our study population included all OV patients from TCGA with available information on Immune immune subtypes (*N* = 234). Gene expression was calculated as log2(x + 1) transformed RSEM normalized counts.

### The prediction of potential drug based on drug-gene correlation

DTP NIC-60 z scores and corresponding RNA-seq composite expression were downloaded from the Cellminer database (https://discover.nci.nih.gov/cellminer/loadDownload.do) [21, 22]. Drug z-score correlated with gene expression and statistically significant (*P* < 0.05) were saved and listed in Table 4. The details of the predicted drug were listed in Table 4. Drug information was derived from the Drugbank database (https://www.drugbank.ca/).

## Results

### Flow chart for the study design

**Fig. 1:**
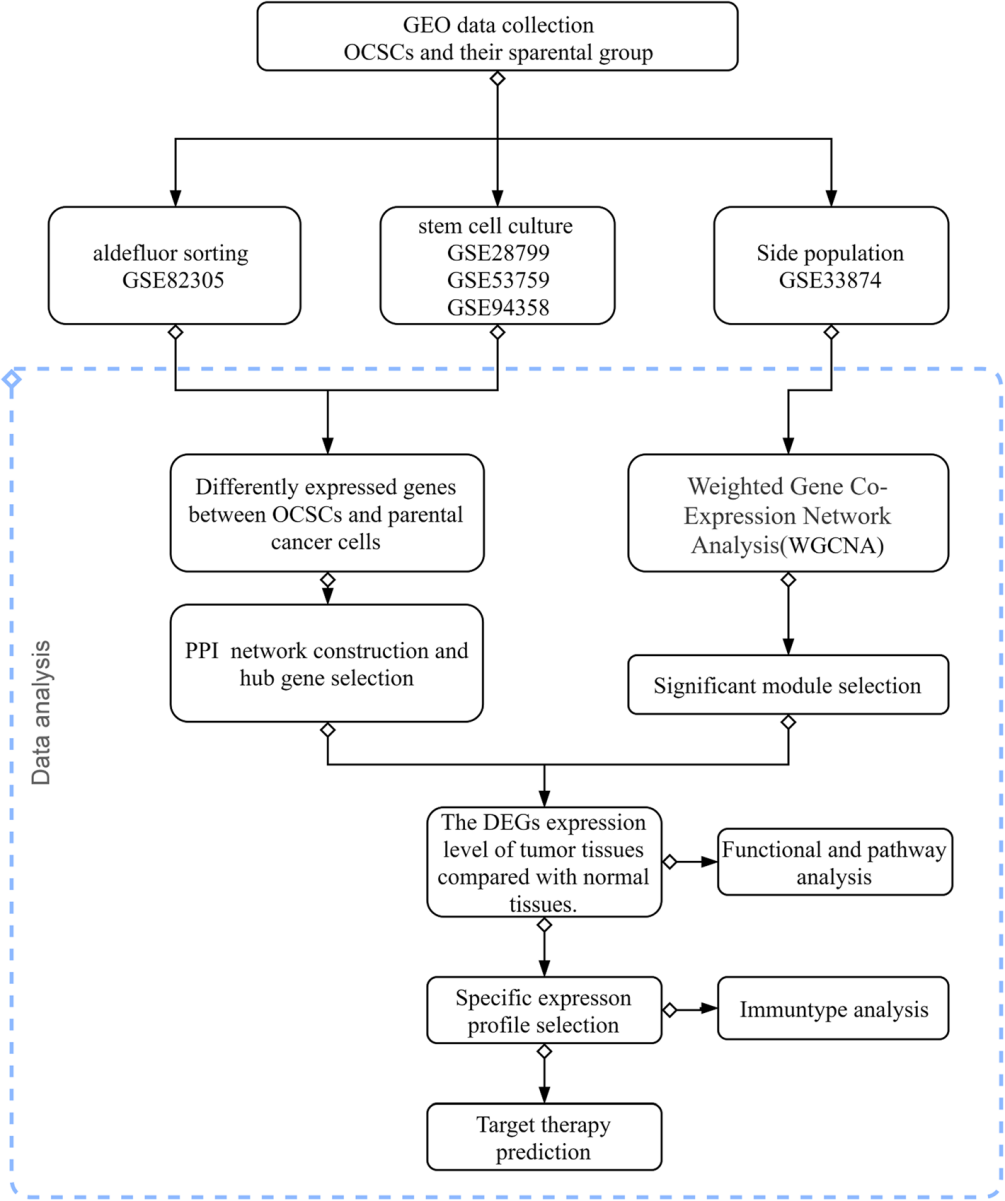
The flow chart for data collecting and data processing in this study

In this study, we conducted a comprehensive analysis of common essential genes in OCSCs isolated by deferent methods and their critical roles in OV by several computational methods. The study design was illustrated in Fig. 1.

### The DEGs among GSE82305, GSE28799, GSE53759 and GSE94358

**Fig. 2:**
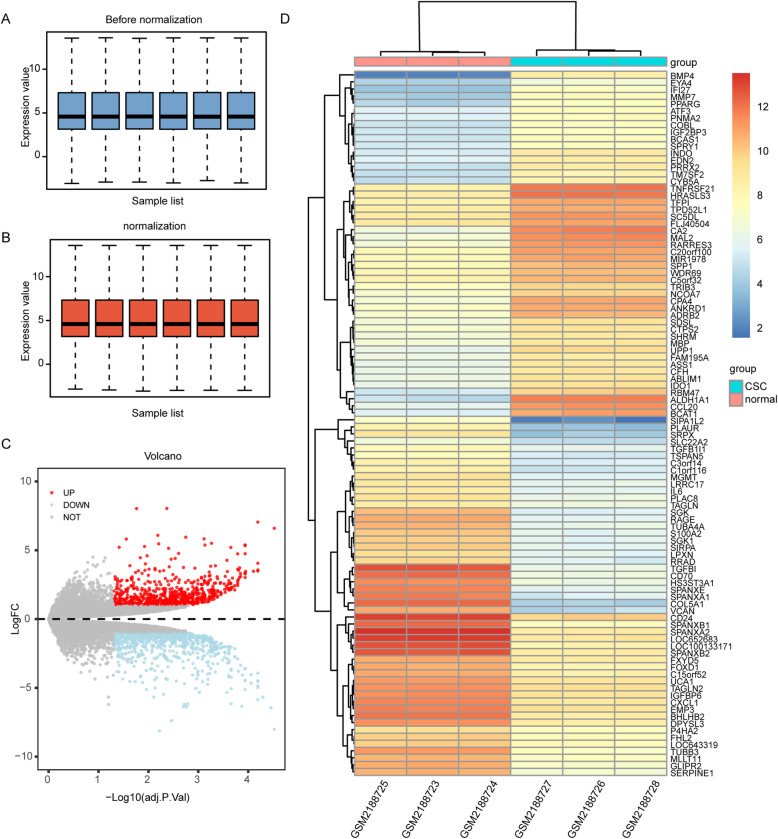
Data processing in the GSE82305 dataset. A-B. Normalization of the GSE82305 dataset. C. The volcano plot showed differentially expressed genes (DEGs) between the two groups of samples in GSE82305. Based on an adjusted *P* < 0.05 and |log fold change| > 1, the red spots represent the upregulated genes and the blue spots represent the downregulated genes; the grey spots represent genes with no significant difference. D. The heatmap of the top 100 DEGs in GSE82305. Orange indicates relative upregulated genes; Blue indicates the relative downregulated gene; yellow suggests no significant change in gene expression

The OCSCs datasets GSE82305, GSE28799, GSE53759 and GSE94358 were normalized. The normalization of GSE82305 was shown in Fig. 2 A and B. The DEGs were selected using the R package ‘limma’ (adjusted *P* < 0.05 and |log fold change (FC)| > 1). The GSE82305 dataset contained 1474 differentially expressed genes, including 724 upregulated genes and 750 downregulated genes (Fig. 2 c). The heatmap of the top 100 genes is shown (Fig. 2 d). The GSE28799 dataset contained 1865 differentially expressed genes, including 959 upregulated genes and 936 downregulated genes (Fig. S1A-B). The GSE53759 dataset contained 273 differentially expressed genes, including 133 downregulated expression genes and 140 downregulated expression genes (Fig. S2A-B). Besides, the GSE94358 dataset contained 305 differential genes, including 50 upregulated genes and 255 downregulated genes (Fig. S3A-B).

### Construction of co-expression networks and identification of key modules

**Fig. 3:**
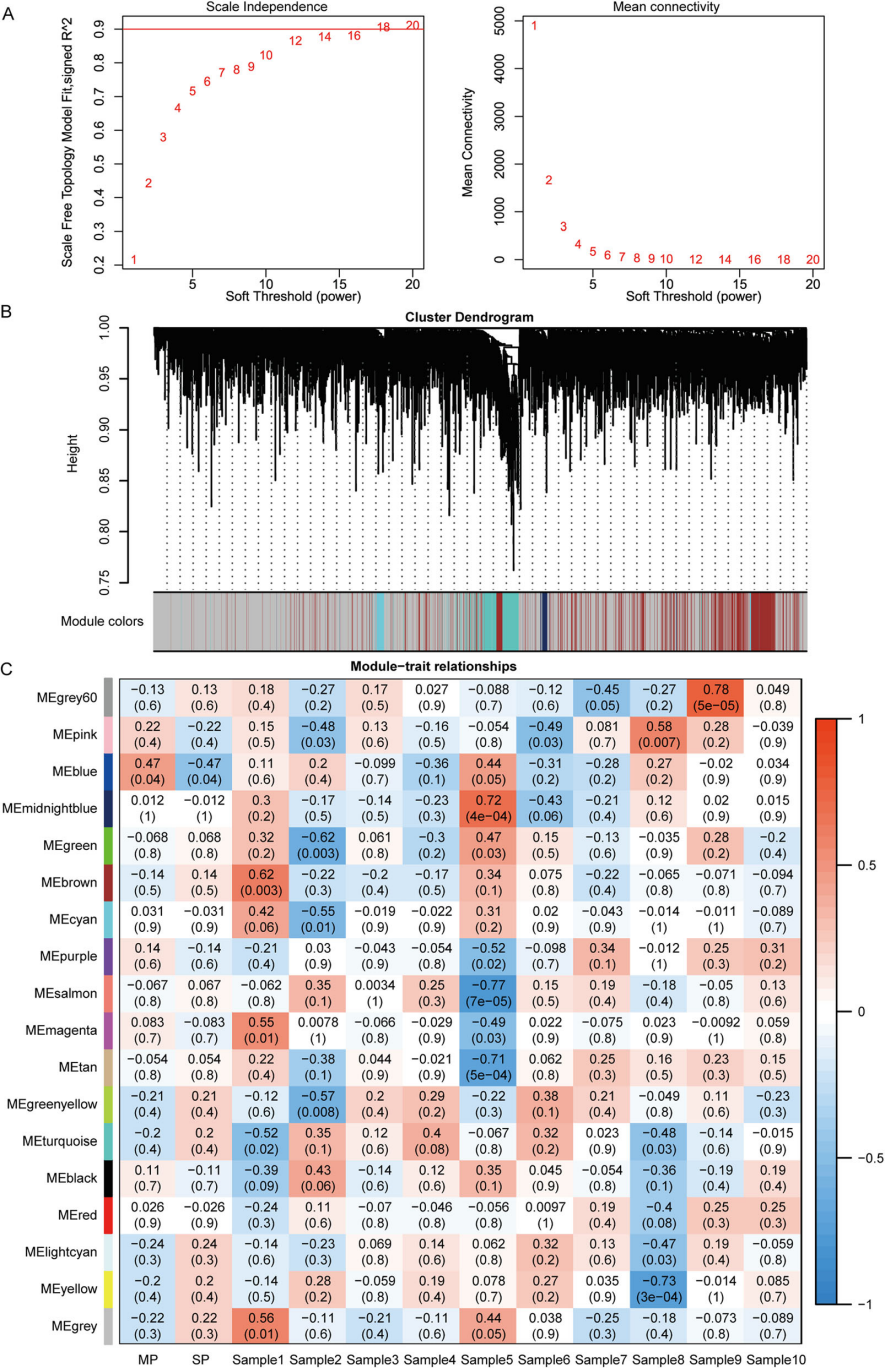
WGCNA for GSE33874. A. Determination of soft-thresholding power in the WGCNA. B. Cluster dendrogram and module assignment for modules from WGCNA. C. Module-sample association relationships. Each row corresponds to a module, labeled by the same color as in (B). The correlation coefficient and *p*-value between the module and the sample or group are shown at the row-column intersection

We employed the WGCNA to analyze the differentially expressed genes between side population (SP) and main population (MP), which were isolated from fresh ascites obtained from 10 women with high-grade serous ovarian adenocarcinoma. The 21,655 genes in 20 samples of the GSE33874 dataset were used to construct the co-expression module. The cluster analysis on these samples and the results were depicted (Fig. S4A). Then, we screened out the soft-thresholding power (Fig. 3 a). When the power value was equal to 16, the independence degree was up to 0.9. Therefore, the power value was used to construct the co-expression module, and the results showed that 18 distinct gene co-expression modules were identified (Fig. 3 b). We analyzed the correlation between module eigengene and group traits and found only one co-expression module significantly correlated with SP and MP (Fig. 3 c). 1154 genes in the blue module correlated negative with SP. We performed PPI network analysis with genes in the blue module. The whole network and the top 3 subnets were depicted (Fig. S4B-C), and its details were listed in Table 2. Subsequently, these genes in the blue module were subjected to GO and KEGG analysis. The top GO term and KEGG pathway in the three subnets were listed in Table S1.

### The integrated DEGs and their function and pathway analysis

**Fig. 4:**
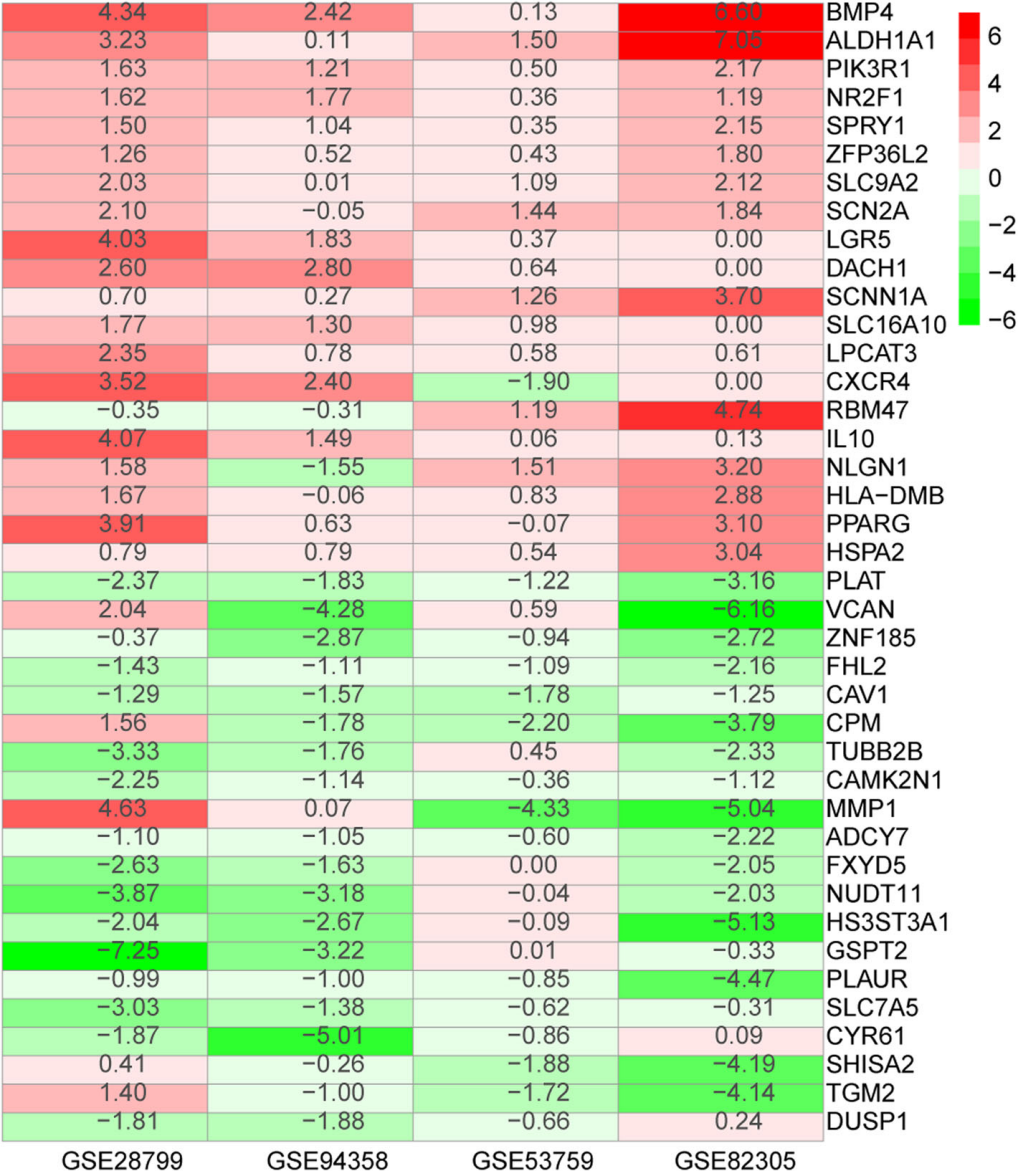
The heatmap of the representative integrated DEGs. The red square represents upregulated DEGs, the green square represents downregulated DEGs, and the value in the square represents the log FC value

**Fig. 5:**
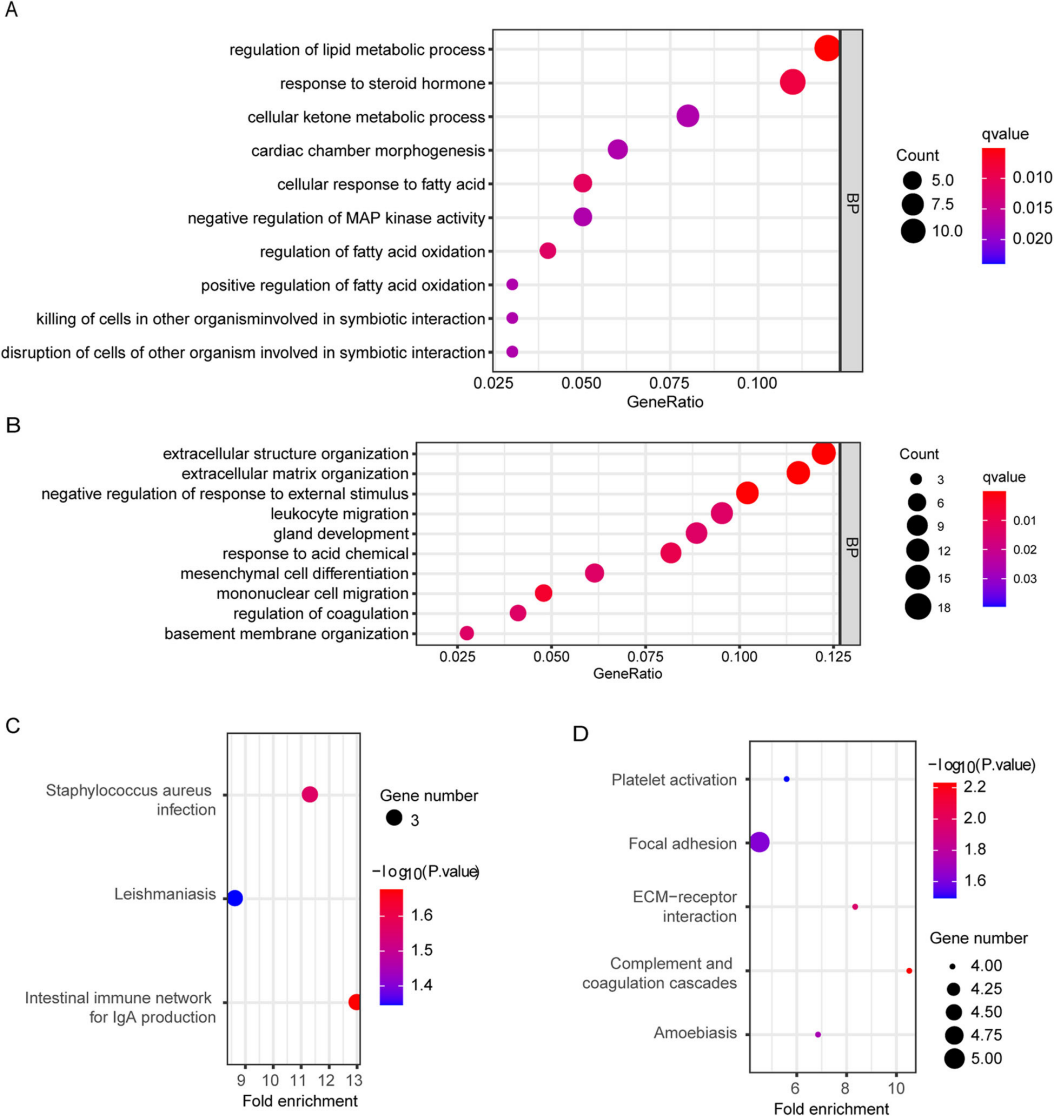
GO and KEGG pathway enrichment analyses of the integrated DEGs. A. GO enrichment analysis of the upregulated integrated DEGs. The top ten BP term with a q-value less than 0.05 were considered as significant and listed. B. GO enrichment analysis of the downregulated integrated DEGs. The top ten BP term with a q-value less than 0.05 were considered as significant and listed. C. The upregulated integrated DEGs were significantly enriched in three KEGG pathways. D. The downregulated integrated DEGs were significantly enriched in five KEGG pathways

**Fig. 6:**
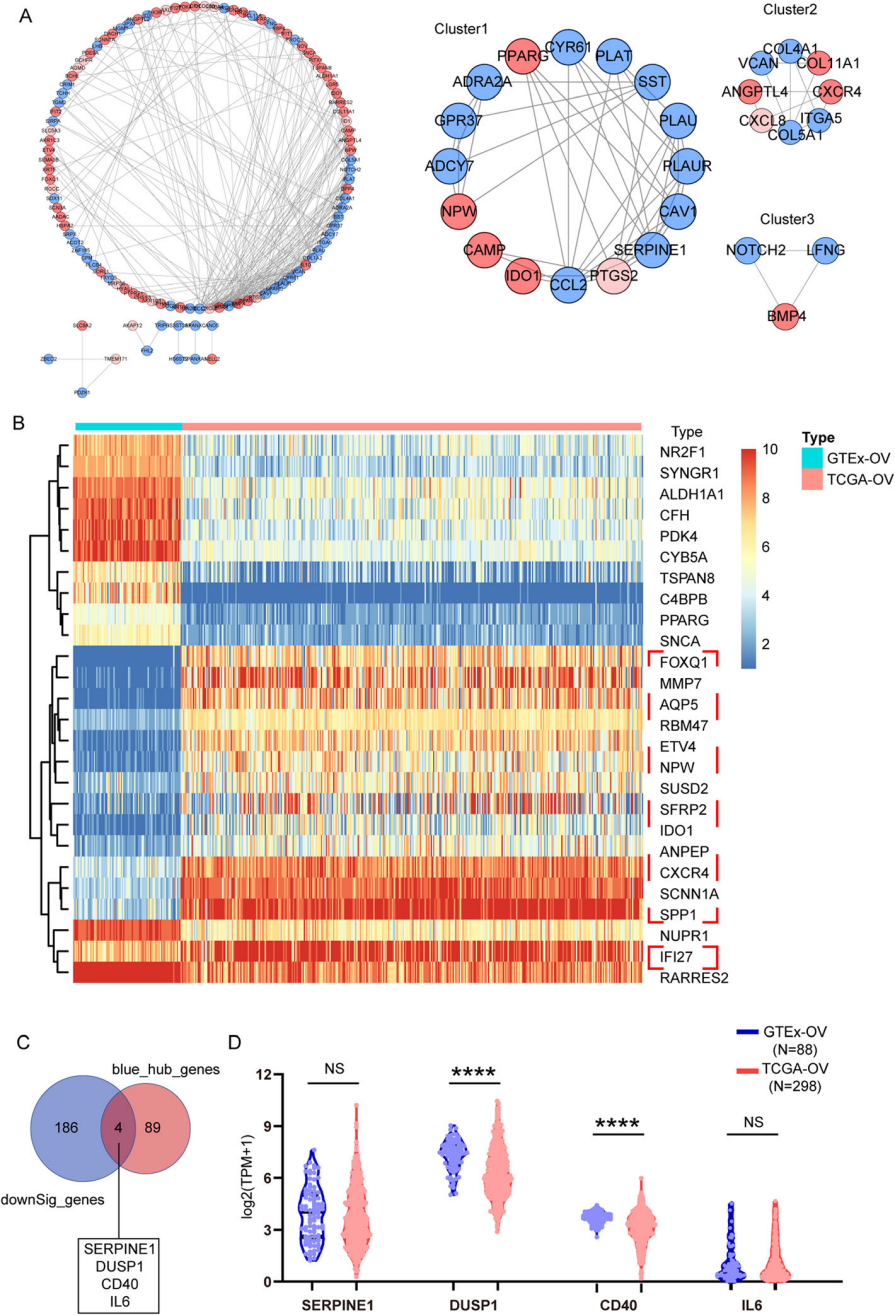
PPI network construction of the integrated DEGs and SEP selection. A. The PPI network on the left was drawn using Cytoscape, and the interaction score was set to medium confidence (0.400). The network nodes represent proteins (red: upregulated proteins and blue: down-regulated proteins), and edges represent protein-protein associations. Three clusters on the right represent the top 3 subnets. B. The gene expression heatmap of the differentially expressed DEGs in TCGA and GTEx. C. Venn plot showing the intersection of the downregulated DEGs and hub genes in the blue module. D. Violin plot showing the expression of indicated genes in TCGA and GTEx (by Kruskal-Wallis test, *P < 0.05, **P < 0.01, ***P < 0.001, ****P < 0.0001)

The DEGs of GSE82305, GSE28799, GSE53759, and GSE94358 datasets were screened using the R package ‘limma’ and sorted according to log FC. The integrated DEGs were obtained using the R package ‘RobustRankAggreg’ (*P* < 0.05, |log FC| > 1). The 343 integrated DEGs, consisting of 111 upregulated genes and 232 downregulated genes, were identified (Table S2). Heat map showing the top 20 upregulated and 20 downregulated genes in the integrated DEGs (Fig. 4). Moreover, these upregulated genes and downregulated genes were subjected to cluster Profiler for GO and KEGG analysis, respectively. GO term annotation showed that these upregulated genes correlated with the regulation of lipid metabolic process, response to steroid hormone, cellular ketone metabolic process, cellular response to fatty acid, regulation of fatty acid oxidation, positive regulation of fatty acid oxidation (BP) (Fig. 5 a). GO analysis also showed that these down-regulated genes related to extracellular structure organization and extracellular matrix organization (Fig. 5b). We figured out that the upregulated genes were mainly enriched in lipid metabolism and the downregulated genes were mainly enriched in extracellular stroma. This indicated that the extracellular matrix regulated cancer stem cell behavior and character to some extent. The upregulated integrated DEGs were mainly enriched in the intestinal immune network for IgA production (Fig. 5c), and downregulated genes in focal adhesion pathways (Fig. 5d). We performed PPI analysis with integrated DEGs. The top 3 subnets were depicted (Fig. 6a), and its details were listed in Table 3.

**Table 3:** The top 3 subnet of DEGs

Cluster	Score (Density*#Nodes)	Nodes	Edges	Node IDs
1	5.6	16	42	ADRA2A, SST, CAV1, GPR37, PPARG, CCL2, PTGS2, CAMP, NPW, ADCY7, IDO1, PLAT, SERPINE1, PLAUR, PLAU, CYR61
2	3.143	8	11	COL4A1, VCAN, ITGA5, ANGPTL4, CXCR4, CXCL8, COL11A1, COL5A1
3	3	3	3	BMP4, LFNG, NOTCH2

### Expression of the integrated DEGs in the ovarian dataset of TCGA and GTEx

We tested the expression of upregulated integrated DEGs in TCGA and GTEx. Among them, 26 genes were differentially expressed (Fig. 6b). We selected the 14 genes, including *FOXQ1, MMP7, AQP5, RBM47, ETV4, NPW, SUSD2, SFRP2, IDO1, ANPEP, CXCR4, SCNN1A, SPP1,* and *IFI27,* which were both overexpressed in OV and OCSCs. Further, we used the Venn diagram to select the shared genes in upregulated integrated DEGs and the genes in the blue module. We obtained four common genes, including *SERPINE1, DUSP1, CD40,* and *IL6* (Fig. 6c), whose expression was similarly low in OV than healthy ovarian tissue (Fig. 6d). In brief, we derived specific expression profile (SEP) of OCSCs, which were composed of *FOXQ1, MMP7, AQP5, RBM47, ETV4, NPW, SUSD2, SFRP2, IDO1, ANPEP, CXCR4, SCNN1A, SPP1* and *IFI27* (upregulated) and *SERPINE1, DUSP1, CD40,* and *IL6* (downregulated).

### SEP expression in immune subtypes

**Fig. 7:**
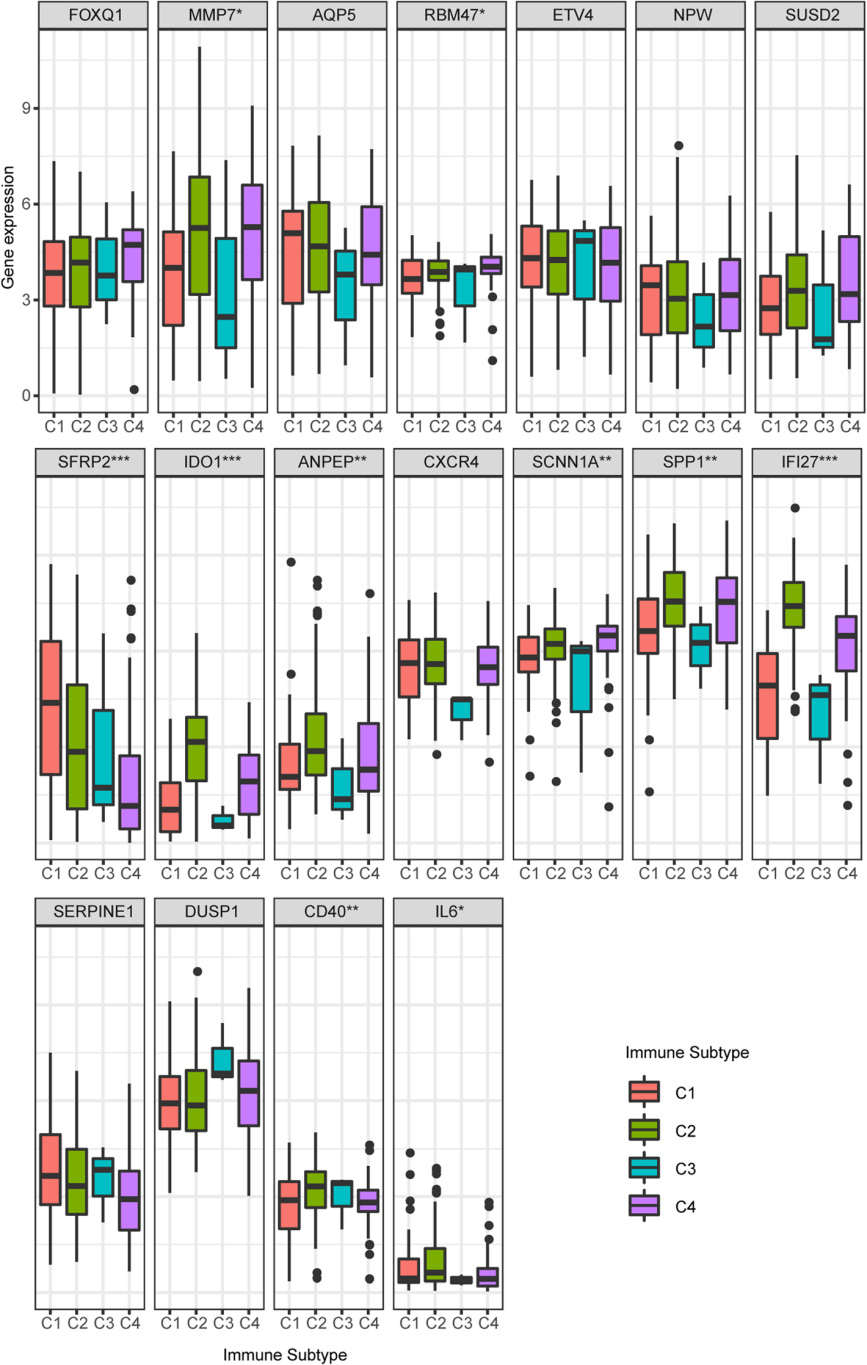
Gene expression of SEP in immune subtypes. Distribution of the mRNA levels of levels for indicated genes with the most significant differences across subtypes in Ovarian cancer (by Kruskal-Wallis test, *P < 0.05, **P < 0.01, ***P < 0.001)

OCSCs can survive from treatment and can be exempt from immunosurveillance. We explored whether the OCSCs signature associated with characteristics contributes to immune escape. A research group had used characteristic immune-oncologic gene signatures to cluster TCGA tumor types into six groups (C1-C6) [20]. The density of specific immune cells and overall prognosis show wide variations between the different immune subtypes. Only 4 immune subtypes were identified in OV, predominantly IFN-gamma Dominant (Immune C2, *N* = 138) and Lymphocyte Depleted (Immune C4, *N* = 53). C2 had the highest M1/M2 macrophage polarization, higher densities of CD8 T cells, a high proliferation rate, and the highest intratumoral heterogeneity. Therefore, we tested the expression of SEP of OCSCs in immune subtypes and found a few upregulated genes had a higher expression in the C2 and C4 group, which were characterized as lymphocyte depleted, may predict the distinct gene profile of cancer stem cells contributes to immune evasion in ovarian Cancer Patients (Fig. 7).

#### Drug susceptibility prediction based on SEP of OCSCs

**Fig. 8:**
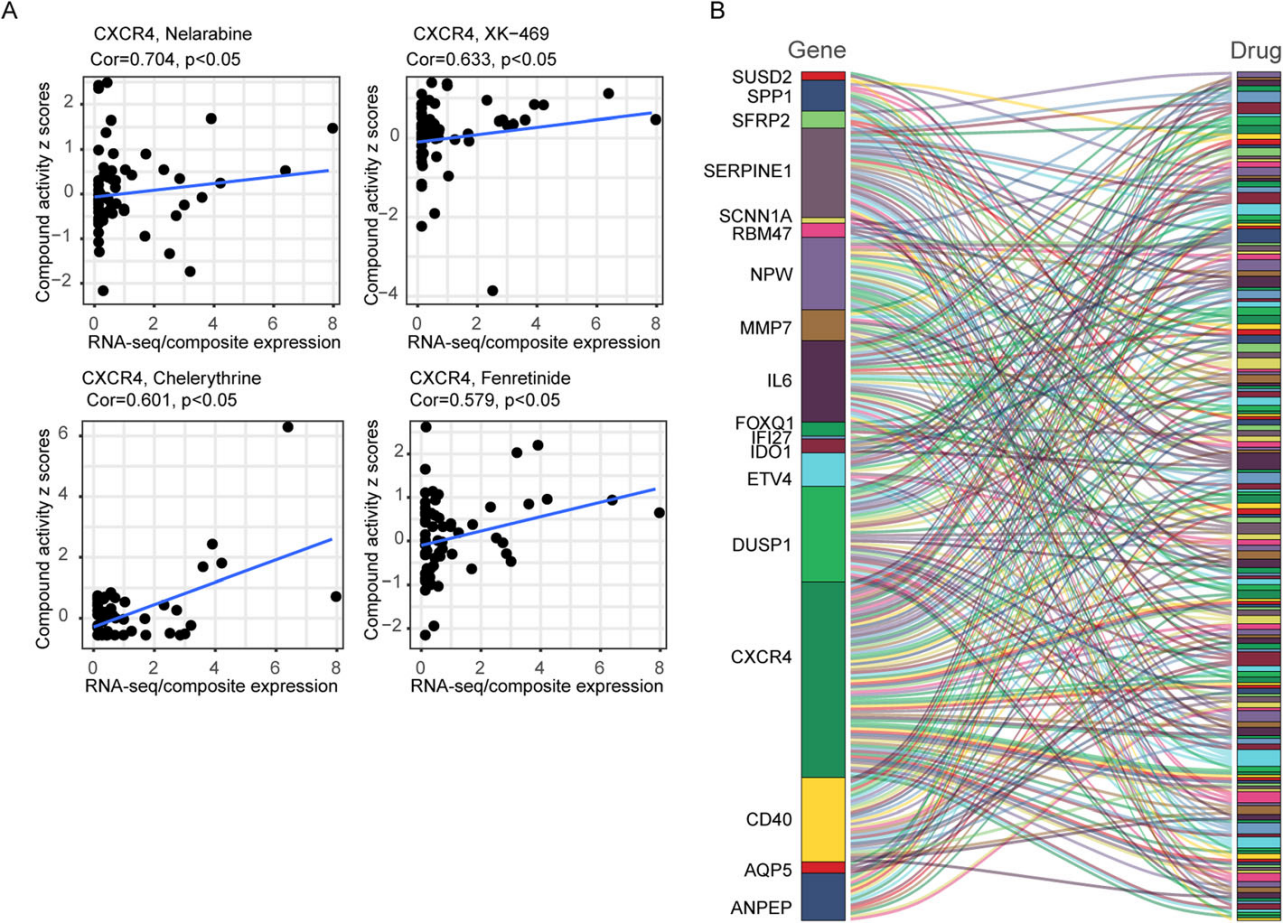
Drug susceptibility prediction based on SEP. A. Representative scatter diagrams show the correlation analysis of gene expression with drug z score (by Pearson correlation test). Each black point represents an independent sample. The blue line is the linear regression. B. Sankey diagram showed the relation of targeted genes and predicted drug

To explore potential molecular-targeted drugs for OCSCs, we download NCI-60 drug z scores and corresponding NCI-60 cell lines RNA-seq/composite expression from the Cellminer database. The higher cell lines z scores have, the more sensitive to the corresponding drug they are. For better applications in the clinic, we employed the US FDA-approved drugs and drugs in clinical trials. We analyzed the correlation between drug z score and SEP. We listed four representative Pearson’s correlation dot plot (Fig. 8 a). All targeted genes and relevant predicted drugs (*P* < 0.05) were shown in the Sankey diagram (Fig. 8 b). We selected drugs that were correlated with at least four target genes as a potential therapy regimen. As a result, we identified ten drugs related to SEP. Detailed information were shown in Table 4. Thus, we inferred that these drugs might be repurposed to the OCSCs with the SEP as drug targets. We extracted drug-indication from DrugBank (https://www.drugbank.ca/) and listed applications of the ten medications in Table 5. For example, Ixabepilone, which was associated with CD40, CXCR4, IL6, and SERPINE1, were used in locally advanced breast cancer and metastatic breast cancer, can potentially be repurposed to treat OCSCs.

**Table 4:** The Person correlation of NCI-60 drug z scores and gene expression

Gene	Drug	cor	*p*value
DUSP1	Bafetinib	−0.43137	0.000579
ETV4		0.330795	0.009836
IL6		− 0.32127	0.012315
SERPINE1		−0.33833	0.008193
CXCR4	Belinostat	0.29389	0.022658
IL6		−0.26566	0.040218
NPW		0.39625	0.001724
SERPINE1		−0.29077	0.024203
DUSP1	Cobimetinib	−0.34957	0.006186
ETV4		0.417929	0.000892
IL6		−0.27774	0.031667
RBM47		0.265132	0.040628
SERPINE1		−0.28517	0.027205
SPP1		0.25733	0.047155
DUSP1	Dabrafenib	−0.29456	0.022338
ETV4		0.427569	0.000656
SERPINE1		−0.30432	0.018078
SPP1		0.278976	0.030886
AQP5	Dolastatin 10	0.382727	0.002544
CD40		−0.54851	5.70E-06
DUSP1		−0.45862	0.000228
IL6		−0.30263	0.018759
SERPINE1		−0.35536	0.005332
CD40	Ixabepilone	−0.26495	0.04077
CXCR4		0.283105	0.028391
IL6		−0.30615	0.017359
SERPINE1		−0.28757	0.025884
DUSP1	Selumetinib	−0.4288	0.00063
ETV4		0.438516	0.000457
FOXQ1		0.285388	0.027085
IL6		−0.29527	0.022002
SERPINE1		−0.3142	0.014492
CD40	Tamoxifen	−0.3044	0.018045
DUSP1		−0.39953	0.001564
IL6		−0.46998	0.000151
SERPINE1		−0.48772	7.73E-05
CD40	Vinorelbine	−0.48333	9.16E-05
DUSP1		−0.3796	0.002777
IL6		−0.29874	0.02042
SERPINE1		−0.28779	0.025769
CXCR4	Vorinostat	0.421015	0.000809
IL6		−0.26497	0.040755
NPW		0.443309	0.000389
SERPINE1		−0.39319	0.001885

**Table 5:** Detailed information of drugs listed in Table 4

Targeted genes	Drugs	Discription	FDA status	Associated Conditions
DUSP1 ETV4 FOXQ1 IL6 SERPINE1	Selumetinib	selumetinib are important tools that can target the problematic overactivity of Raf-MEK-ERK signaling pathway.	Approved, Investigational	NA
AQP5 CD40 DUSP1 IL6 SERPINE1	Dolastatin 10	Dolastatin 10 has been used in trials studying the treatment of Sarcoma, Leukemia, Lymphoma, Liver Cancer, and Kidney Cancer, among others.	Investigational	NA
CD40 CXCR4 IL6 SERPINE1	Ixabepilone	Ixabepilone is an epothilone B analog developed by Bristol-Myers Squibb as a cancer drug.	Approved, Investigational	Locally Advanced Breast Cancer (LABC) Metastatic Breast Cancer
CXCR4 IL6 NPW SERPINE1	Vorinostat	Vorinostat (rINN) or suberoylanilide hydroxamic acid (SAHA), is a drug currently under investigation for the treatment of cutaneous T cell lymphoma (CTCL)	Approved, Investigational	Persistent Cutaneous T-Cell Lymphoma Progressive Cutaneous T-cell lymphoma Recurrent Cutaneous T-cell lymphoma
	Belinostat	Belinostat is a novel agent that inhibits the enzyme histone deacetylase (HDAC) with a sulfonamide-hydroxamide structure.	Approved, Investigational	Relapsed Peripheral T-Cell Lymphoma Refractory Peripheral T-cell Lymphoma Unspecified
CD40 DUSP1 IL6 SERPINE1	Vinorelbine	Vinorelbine is an anti-mitotic chemotherapy drug that is used in the treatment of several types of malignancies	Approved, Investigational	Advanced Non Small Cell Lung Cancer Esophageal Cancers Locally Advanced Non-Small Cell Lung Cancer Metastatic Breast Cancer Recurrent Cervical Cancer Soft Tissue Sarcoma (STS) Recurrent, IV-B Cervical cancer
	Tamoxifen	Tamoxifen is a non-steroidal antiestrogen used to treat estrogen receptor positive breast cancers as well as prevent the incidence of breast cancer in high risk populations	Approved	Breast Cancer Desmoid Tumors Endometrial Cancer Gynecomastia Invasive Breast Cancer Invasive Breast Carcinoma Metastatic Breast Cancer Ovarian Cancer Puberty, Precocious
CD40 CXCR4 DUSP1 SERPINE1	Raloxifene	Raloxifene is a second generation selective estrogen receptor modulator (SERM) that mediates anti-estrogenic effects on breast and uterine tissues, and estrogenic effects on bone, lipid metabolism, and blood coagulation.	Approved, Investigational	Invasive Breast Cancer Osteoporosis Osteoporosis caused by glucocorticoid
DUSP1 ETV4 IL6 SERPINE1	Tanespimycin	Tanespimycin, manufactured by Conforma Therapeutics is under development as a small molecule inhibitor of heat shock protein 90 (HSP90). It is developed for the treatment of several types of cancer, solid tumors or chronic myelogenous leukemia.	Investigational	NA
DUSP1 ETV4 SERPINE1 SPP1	Dabrafenib	Dabrafenib mesylate (Tafinlar) is a reversible ATP-competitive kinase inhibitor and targets the MAPK pathway.	Approved, Investigational	Metastatic Melanoma Unresectable Melanoma

## Discussion

Most ovarian cancer patients respond to initial chemotherapy, but more than 70% of patients will develop tumor recurrence and eventually develop resistance to treatment [2]. Ovarian cancer stem cells are thought to promote the recurrence of ovarian cancer and lead to the development of treatment resistance [23]. There are several methods to isolate ovarian tumor stem cells. However, the mechanisms of ovarian tumor stem cells obtained by different routes to promote tumor development are not the same. The core signaling pathways that regulate ovarian cancer stem cells remain unclear. There is still lacking in effective drugs and drug combinations to eliminate them to improve cancer survival.

In this study, the GSE82305, GSE28799, GSE53759, and GSE94358 datasets were analyzed, and 343 integrated DEGs were found. As for study GSE33874, we applied WGCNA analysis and got 18 significant enriched modules. The blue module was significantly correlated with SP and MP. The 343 integrated DEGs were then subjected to gene enrichment analysis. The upregulated genes were mainly enriched in lipid metabolism, and the downregulated genes were enriched primarily in extracellular structure organization. The KEGG pathway analysis revealed that these downregulated integrated DEGs are mostly enriched in focal adhesion, which is essential in the extracellular matrix formation. Like the metabolic characteristics of tumor cells, rapidly proliferating stem cells mainly rely on aerobic glycolysis to provide energy [14]. In a study, chemical imaging of a single living cell was performed. They identified and described lipid unsaturation in ovarian cancer stem cells for the first time and suggested to effectively eliminate CSCs by inhibition of lipid desaturases [24]. OXPHOS pathway and lipid metabolism in cancer stem cells are recognized as targets for the development of novel anticancer therapies [25]. In our study, we found upregulated genes were enriched in lipid metabolism, which suggested cellular event accumulation of lipids and secondary metabolites. To meet the increasing energetic requirements of CSCs, the lipid metabolic pathway can flexibly turn to the other metabolic pathways [26]. CSCs are incredibly reliant on the activity of enzymes involved in lipid metabolism, which engaged in CSCs fate decisions, such as Hippo and Wnt signal pathway [25]. Emerging evidence suggests that alterations in lipid- and fatty acid-associated pathways are essential for the maintenance of CSCs [27, 28]. Some recent evidence has demonstrated that cancer stem cell maintenance and differentiation is regulated by extracellular matrix mechanics [29, 30]. Interactions of cells with the extracellular matrix are crucial for the establishment and maintenance of stem cell [31, 32]. Our results also confirm that the most significant GO term in subnet3 of the blue module is response to mechanical stimulus (Table S1). In our study, we uncovered the changes in lipid metabolism and extracellular matrix are universal, independent of cell types and sorting methods. This phenomenon addresses the interactions of OCSCs with environment which result in the modulation of lipid metabolism, and thereby of OCSCs phenotype.

Studies have shown that tumor stem cells can reduce and evade from NK cells by downregulating the active ligand of NK cells, such as major histocompatibility I polypeptide related sequences A (MICA) and histocompatibility I polypeptide related sequences B (MICB), so as to escape from immune surveillance [33]. The cancer stem cells have been identified to survived within a specialized cellular niche through the crosstalk with the surrounding microenvironment [34]. Ovarian carcer was classified into four immune subtypes (C1-C4) based on the study of Thorsson et al. C2 and C4 showed poor prognosis, despite C2 had a substantial immune component. Our results showed the expression of MMP7, RBM47, and SCNN1A were significantly higher in C2 and C4 than other immune subtypes. In this regard, the complex interrelations between cancer stem cells and tumor immune microenvironment might play an vital role in MPM tumorigenesis. To identify potential drugs for OCSCs based on the SEP, we compared drug sensitivity of US FDA-approved anticancer drugs, which can be conducive to treatment. We chose ten possible drugs for OCSCs. These drugs have been used in other kinds of diseases and are believed to be further explored in tumor stem cell treatment.

In summary, the purpose of this study was to increase our understanding of the core signal pathway in OCSCs through an integrated bioinformatics analysis that aimed to identify integrated DEGs and the related pathways enriched in OCSCs. Our research also identified SEP that could serve as biomarkers and therapeutic targets of OCSCs. However, further research is required to establish the therapeutic efficiency of the potential drugs.

## Supplementary information

**Additional file 1 Figure S1.** Data processing in the GSE28799 dataset. A. The volcano plot showed differentially expressed genes (DEGs) between the two groups of samples in GSE28799. Based on an adjusted *P* < 0.05 and |log fold change| > 1, the red spots represent the upregulated genes and the blue spots represent the downregulated genes; the grey spots represent genes with no significant difference. B. The heatmap of the top 100 DEGs in GSE28799. Orange indicates relative upregulated genes; Blue indicates the relative downregulated gene; yellow suggests no significant change in gene expression;

**Additional file 2 Figure S2.** Data processing in the GSE53759 dataset. A. The volcano plot showed differentially expressed genes (DEGs) between the two groups of samples in GSE53759. Based on an adjusted P < 0.05 and |log fold change| > 1, the red spots represent the upregulated genes and the blue spots represent the downregulated genes; the grey spots represent genes with no significant difference. B. The heatmap of the top 100 DEGs in GSE53759. Orange indicates relative upregulated genes; Blue indicates the relative downregulated gene; yellow suggests no significant change in gene expression.

**Additional file 3 Figure S3.** Data processing in the GSE94358 dataset. A. The volcano plot showed differentially expressed genes (DEGs) between the two groups of samples in GSE94358. Based on an adjusted P < 0.05 and |log fold change| > 1, the red spots represent the upregulated genes and the blue spots represent the downregulated genes; the grey spots represent genes with no significant difference. B. The heatmap of the top 100 DEGs in GSE94358. Orange indicates relative upregulated genes; Blue indicates the relative downregulated gene; yellow suggests no significant change in gene expression.

**Additional file 4 Figure S4.** PPI networks analysis of the blue module. A. Cluster dendrogram of 20 samples in GSE33874. B. PPI networks of genes in the blue module. C. The top 3 subnets of network in (B).

**Additional file 5 Table S1.**

**Additional file 6 Table S2.**

## Data Availability

The datasets analyzed during the current study are available in GEO and TCGA database.
