# Supplementary figures and images for: Integrative analysis of the common genetic characteristics in ovarian cancer stem cells sorted by multiple approaches
Source: J Ovarian Res. 2020 Sep 25;13:116. doi: 10.1186/s13048-020-00715-7 (PMC7519480; doi:10.1186/s13048-020-00715-7)

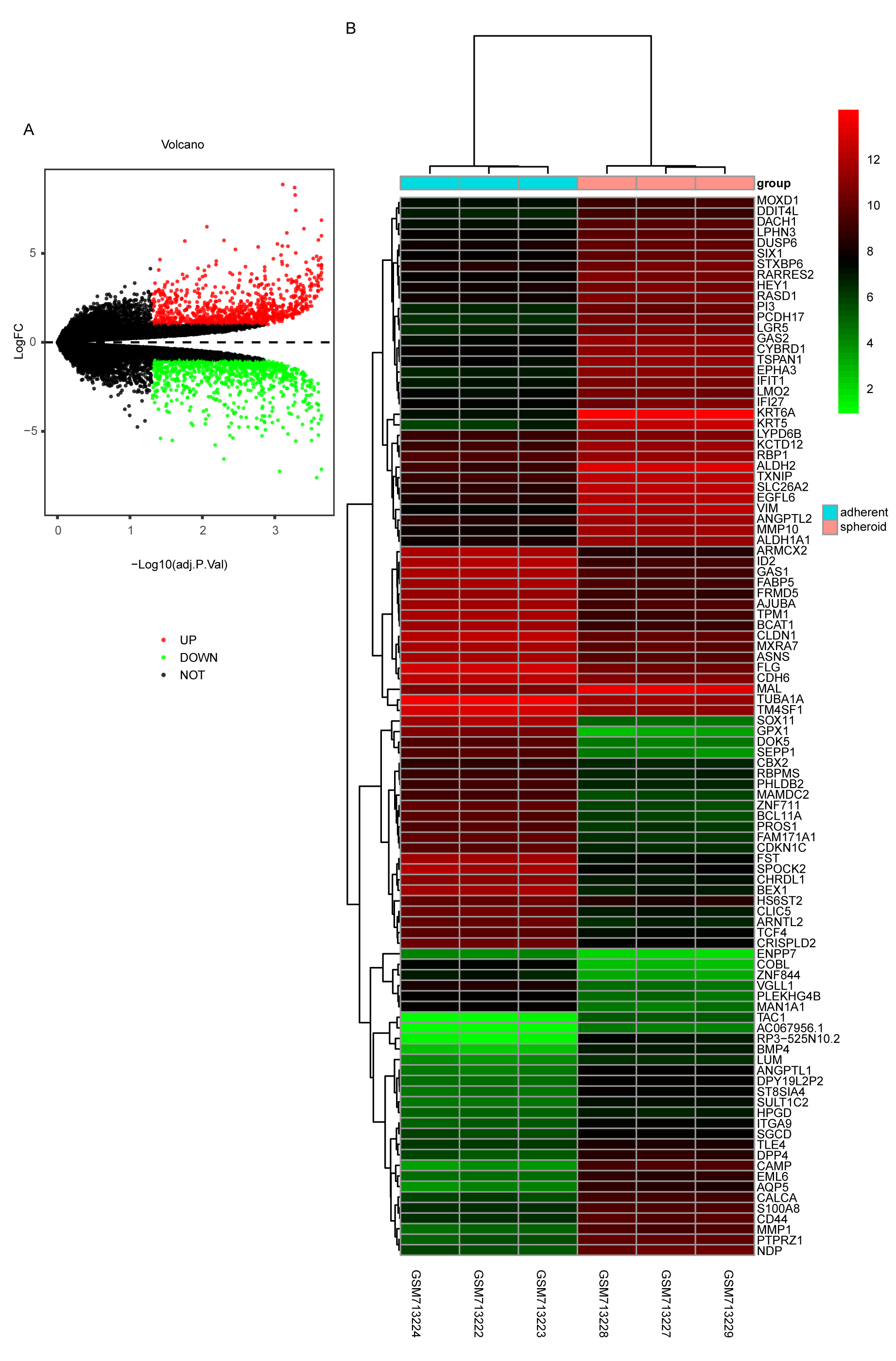

Supplement: Supplementary file 1 — Additional file 1 Figure S1. Data processing in the GSE28799 dataset. A. The volcano plot showed differentially expressed genes (DEGs) between the two groups of samples in GSE28799. Based on an adjusted P < 0.05 and |log fold change| > 1, the red spots represent the upregulated genes and the blue spots represent the downregulated genes; the grey spots represent genes with no significant difference. B. The heatmap of the top 100 DEGs in GSE28799. Orange indicates relative upregulated genes; Blue indicates the relative downregulated gene; yellow suggests no significant change in gene expression; [file 13048_2020_715_MOESM1_ESM.tif]

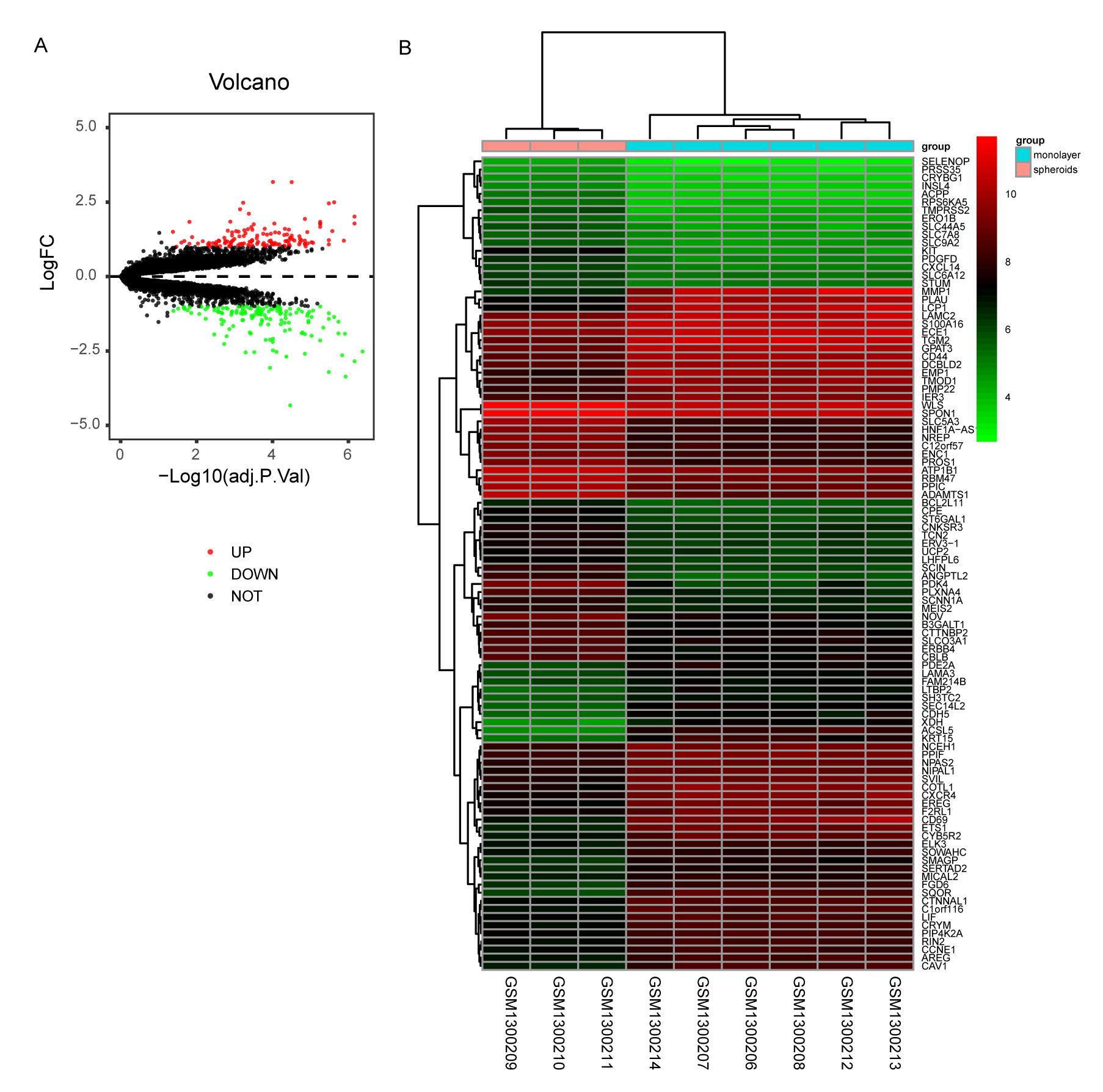

Supplement: Supplementary file 2 — Additional file 2 Figure S2. Data processing in the GSE53759 dataset. A. The volcano plot showed differentially expressed genes (DEGs) between the two groups of samples in GSE53759. Based on an adjusted P < 0.05 and |log fold change| > 1, the red spots represent the upregulated genes and the blue spots represent the downregulated genes; the grey spots represent genes with no significant difference. B. The heatmap of the top 100 DEGs in GSE53759. Orange indicates relative upregulated genes; Blue indicates the relative downregulated gene; yellow suggests no significant change in gene expression. [file 13048_2020_715_MOESM2_ESM.tif]

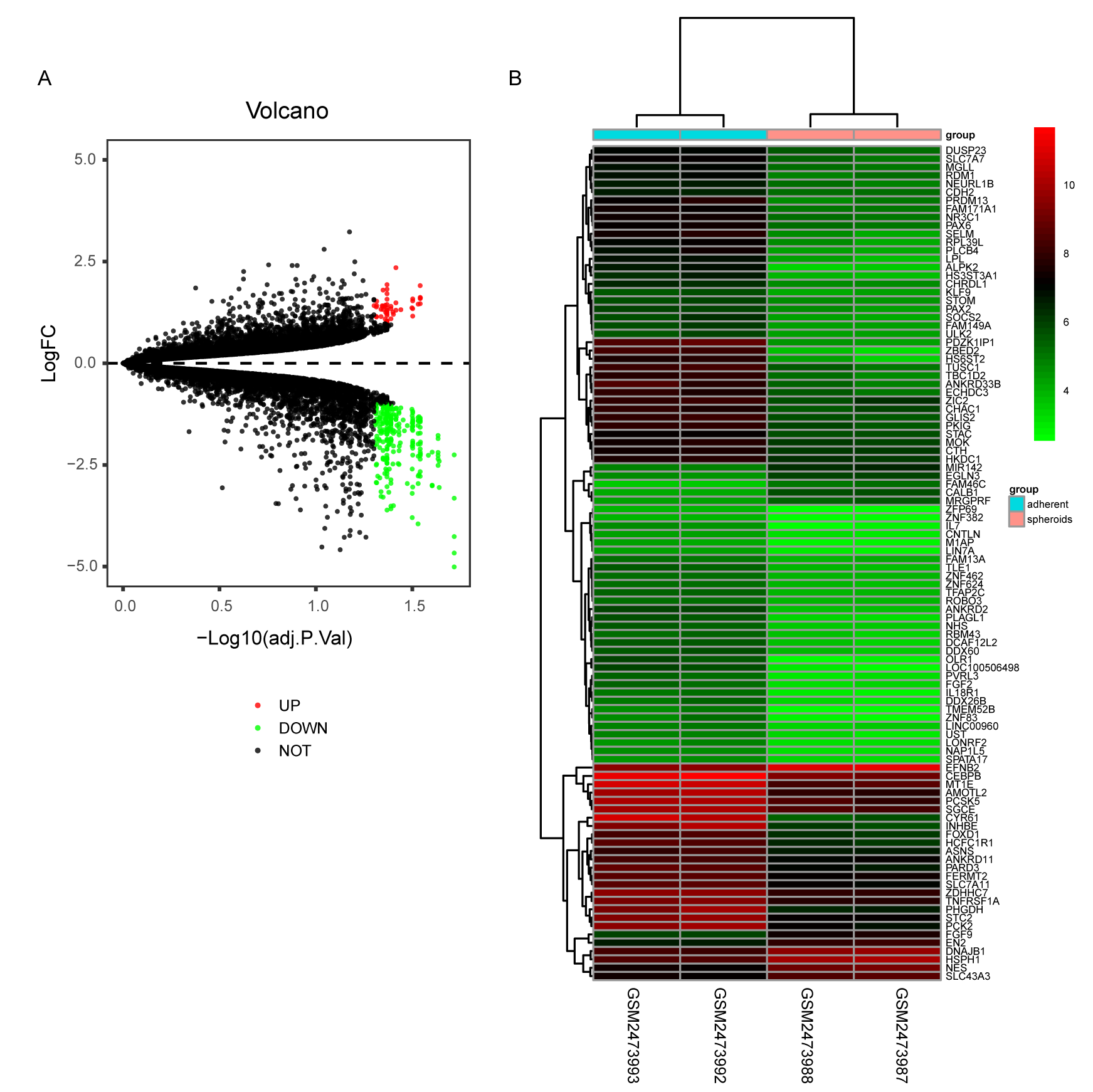

Supplement: Supplementary file 3 — Additional file 3 Figure S3. Data processing in the GSE94358 dataset. A. The volcano plot showed differentially expressed genes (DEGs) between the two groups of samples in GSE94358. Based on an adjusted P < 0.05 and |log fold change| > 1, the red spots represent the upregulated genes and the blue spots represent the downregulated genes; the grey spots represent genes with no significant difference. B. The heatmap of the top 100 DEGs in GSE94358. Orange indicates relative upregulated genes; Blue indicates the relative downregulated gene; yellow suggests no significant change in gene expression. [file 13048_2020_715_MOESM3_ESM.tif]

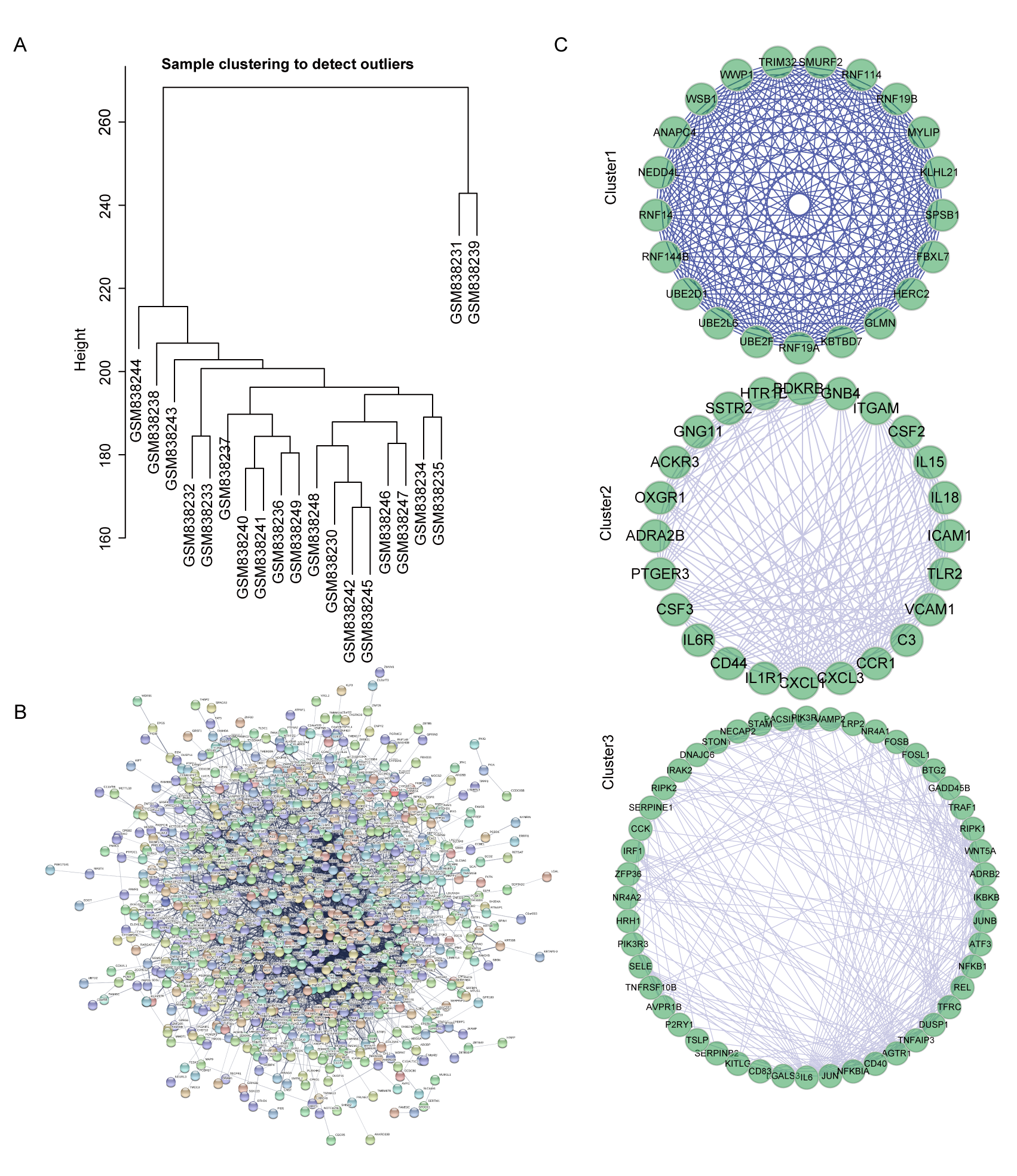

Supplement: Supplementary file 4 — Additional file 4 Figure S4. PPI networks analysis of the blue module. A. Cluster dendrogram of 20 samples in GSE33874. B. PPI networks of genes in the blue module. C. The top 3 subnets of network in (B). [file 13048_2020_715_MOESM4_ESM.tif]
